# Improving the Quality of General Surgical Operation Notes According to the Royal College of Surgeons (RCS) Guidelines: A Closed-Loop Audit

**DOI:** 10.7759/cureus.48147

**Published:** 2023-11-02

**Authors:** Hamza Khan Toru, Muhammad Aizaz, Abdullah A Orakzai, Zaka Ullah Jan, Ahmad Ammar Khattak, Danyal Ahmad

**Affiliations:** 1 Department of General Surgery, Khyber Teaching Hospital, Peshawar, PAK; 2 Department of General Medicine, Russells Hall Hospital, Dudley Group NHS Foundation Trust, Birmingham, GBR; 3 Department of Internal Medicine, Rochester Regional Health, Rochester, USA; 4 Department of General Medicine, Kabir Medical College, Gandhara University, Peshawar, PAK; 5 Department of General Medicine, Khyber Teaching Hospital, Peshawar, PAK

**Keywords:** patient care, surgical records, documentation, rcs guidelines, operative notes

## Abstract

Background

Thorough and precise operative notes play a vital role in patient care, facilitating communication among healthcare teams and serving as essential documents for legal purposes. Poor documentation can jeopardize patient safety and the quality of care provided. The use of standardized guidelines, such as those endorsed by recognized surgical organizations, is crucial to ensure consistent and detailed record-keeping. This study aims to assess the alignment of postoperative notes with established guidelines, with the goal of enhancing documentation practices in the healthcare setting.

Objectives

This study aimed to evaluate the quality and comprehensiveness of postoperative surgical notes and assess their alignment with established guidelines for surgical documentation, specifically focusing on adherence to recognized standards in surgical practice.

Methods

This cross-sectional audit assessed 150 operative notes (79 pre-implementation and 71 post-implementation of the Royal College of Surgeons (RCS) guidelines) in the General Surgery Unit at Khyber Teaching Hospital Peshawar, Pakistan. Data included peri-operative findings, operative diagnosis, team information, operational details, complications, procedures, prosthesis, closure, DVT prophylaxis, time out, postoperative orders, and signatures.

Results

Post-implementation, peri-operative findings were noted in 68 (95.7%) notes, compared to 56 (70.8%) pre-implementation. Operative diagnosis consistently increased from 65 (82.3%) to 69 (97.2%). Post-implementation, operation type, date, and time were consistently included in 67 (94.4%) notes. Complications, additional procedures, and tissue alterations surged to 66 (92.9%), 64 (90.1%), and 60 (84.5%), respectively. Prosthesis and closure techniques were recorded in 65 (91.5%) and 66 (92.9%). Deep vein thrombosis (DVT) prophylaxis and "time out" were documented in 68 (95.8%) notes. Postoperative orders and signatures improved to 70 (98.6%) and 69 (97.2%), respectively.

Conclusion

Our study revealed the significant positive impact of RCS guideline implementation on operative note documentation. Improvements were noted in essential components such as peri-operative findings, diagnosis, team details, complications, procedures, and more. These enhancements have far-reaching implications, bolstering patient care and ensuring clear communication among healthcare providers, all while serving a vital role in medico-legal matters. By adopting the RCS guidelines, healthcare institutions commit to a higher documentation standard, ultimately supporting good clinical governance.

## Introduction

Operative notes play a pivotal role in providing a comprehensive account of surgical procedures [1]. These meticulously crafted records are indispensable for the immediate postoperative management of patients and long-term follow-up care. Furthermore, within the intricate web of healthcare delivery, medical records serve as a crucial conduit for communication among different departments within a healthcare facility, ensuring the seamless continuity of care and efficient time management [2]. The quality of operative notes holds profound significance as it directly influences decision-making in patient management, ultimately shaping the overall performance of surgical specialties. Inadequate or poorly composed postoperative notes can result in errors during postoperative care, compromise patient well-being, and protract hospital admissions [3]. These avoidable issues underscore the critical importance of proper documentation practices. Furthermore, precise and detailed medical records are pivotal beyond immediate patient care. They serve as invaluable assets for audit purposes and as essential components of research projects [4]. Ultimately, their meticulous upkeep contributes to an elevated standard of patient care, which stands as the central goal of every healthcare institution. Operative notes, in particular, hold substantial relevance in medico-legal contexts. They serve as potent tools for resolving inconsistencies or disputes [5]. Comprehensive and meticulously maintained notes serve as evidence, substantiating and confirming facts when needed. The legibility and clarity of postoperative notes are of paramount importance to ensure the effective management of patients. Regrettably, many residency training programs worldwide have overlooked the significance of training residents in the art of composing high-quality operative documentation [6]. It is imperative to equip residents with the skills and knowledge required to adhere to established guidelines for postoperative notes, ultimately enhancing the quality of healthcare delivery. The adoption of standardized proforma for operative notes represents a noteworthy step toward achieving impeccable record-keeping. In many regions across the globe, operative notes are predominantly handwritten, raising concerns about legibility [7]. The transition to typed notes has demonstrated the potential to enhance documentation quality while reducing errors. Moreover, computerized notes offer a plethora of advantages over handwritten ones, including improved legibility, more efficient time management, and seamless communication between different healthcare facility departments. However, this transition necessitates substantial resources, encompassing staff training and the installation of computer infrastructure. The Royal College of Surgeons guidelines, outlined in "Good Surgical Practice" published in 2014, have emerged as the gold standard for operative notes. These guidelines delineate eighteen essential parameters for operative notes documentation, offering clarity and conciseness that are universally applicable across various surgical departments. Adherence to these guidelines signifies a commitment to elevating the quality of operative documentation, thus contributing to the broader principles of good clinical governance. In light of the aforementioned considerations, the primary objective of this study is to evaluate the alignment of postoperative notes within our hospital with the RCS guidelines. Additionally, the study aims to identify any deficiencies in the documentation of these critical records, ultimately striving to enhance the quality of patient care through improved documentation practices.

## Materials and methods

This cross-sectional audit for enhanced operative notes study focused on evaluating the quality of operative notes, both before and after the implementation of improved documentation practices in accordance with the RCS guidelines. The study was conducted after obtaining approval from the hospital's ethical and research committee under reference # 546-2 in the General Surgery Unit at Khyber Teaching Hospital, Peshawar. This clinical audit study spanned a total of six months, divided into two phases: a three-month pre-implementation phase, i.e., July to September 2022, and a three-month post-implementation phase (October to December 2022). 

Inclusion criteria

Operative notes of patients who underwent any surgical procedure within the general surgery units of KTH were included. Both male and female patients were considered. Each note was given a legibility score that ranged from "highly legible" to "poor legibility." Inter-rater reliability was maintained, and the assessment was conducted in a blinded manner, ensuring impartiality and consistency in the evaluation. This rigorous approach facilitated an objective determination of note legibility, a vital component of our study. 

Exclusion criteria

**Figure 1:**
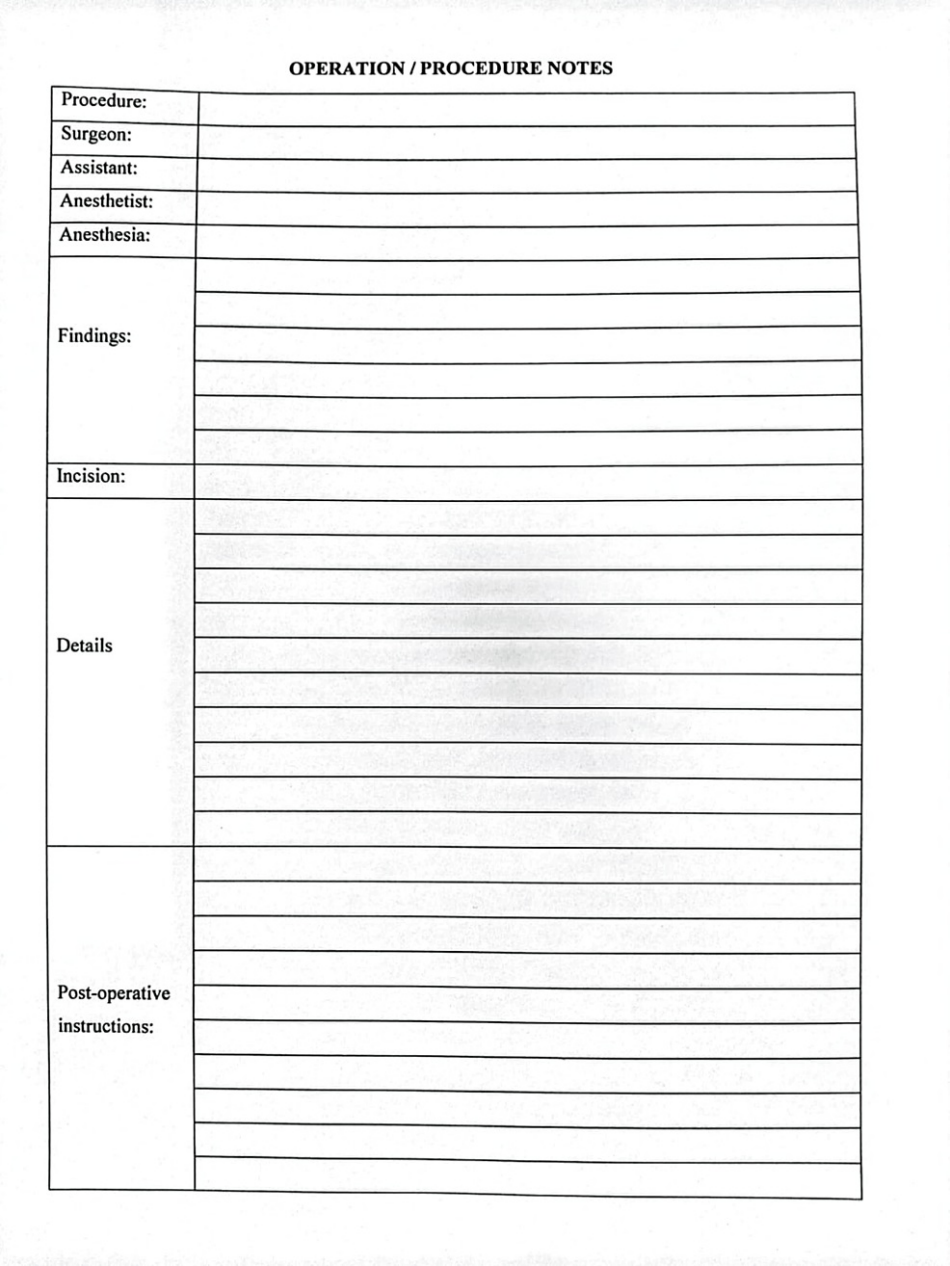
Operative notes before the implementation of RCS guidelines. RCS: Royal College of Surgeons.

Operative notes from patients in units other than general surgery were excluded, as were minor surgical procedures carried out under local anesthesia. This is because minor procedures often involve different documentation requirements compared to major surgical procedures. By focusing only on major surgical procedures, we could concentrate on the specific documentation needs that typically involve more comprehensive and complex operative notes. Also excluded were illegible operative notes. In the preliminary phase, which spanned the first three months, the study incorporated all general surgical cases meeting the inclusion criteria, amounting to a total of 79 cases. Subsequently, during the ensuing three-month post-implementation phase, we consistently applied the same methodology, leading to the inclusion of all cases that satisfied the predetermined inclusion criteria. As a result, the study encompassed a collective enrollment of 71 cases. The post-implementation phase consisted of full implementation of the RCS-aligned proforma; systematic data collection using the proforma; rigorous data analysis; comparative evaluation; expert assessment of adherence to RCS guidelines; quantitative representation of findings; statistical analysis using SPSS; strict adherence to data confidentiality; and comprehensive documentation of results. Operative notes that met the inclusion criteria were included in the study. Detailed information about the various components of the operative notes was systematically recorded, and any deficiencies were noted, as shown in Figure 1.

After data collection in the first stage of the audit cycle, a comprehensive analysis was conducted to assess the alignment of our existing operative notes practices with the guidelines established by the RCS. An educational intervention meeting was subsequently called where members from all surgical wards were present, and the points were discussed in detail, including preparatory planning, presentation of pre-implementation findings, discussion of identified deficiencies, attribution of deficiencies, introduction of the RCS-aligned proforma, training and familiarization, opportunity for questions and clarifications, emphasis on consistency, and quality and post-implementation phase preparation. It was observed that there was very poor documentation of many of the aspects mentioned by the RCS, and it was mainly attributed to the deficient nature of the operative notes proforma that was currently being used. Therefore, a new and improved proforma was developed. The development of the proforma involved a thorough process. We began with a comprehensive literature review to understand RCS guidelines and best practices. The proforma was meticulously designed to include key components from the RCS guidelines. Multiple drafts were created, incorporating feedback, and a pilot phase was conducted for usability testing. The final proforma was approved by the hospital's ethical and research committee, and its use was implemented for documenting post-operative notes in all general surgical theatres. The first three months constituted the pre-implementation phase. A non-probability (consecutive) sampling technique was employed, and data were collected using a semi-structured questionnaire. A panel of experts comprising experienced healthcare providers such as surgeons, anesthetists, and nurses alongside surgical experts with specialized knowledge in specific domains reviewed the operative notes for adherence to the RCS guidelines outlined in "Good Surgical Practice," published by RCS England in 2014. Each guideline area was marked as 'Y' for Yes or 'N' for No. The collected data were summarized using frequencies and percentages. The same data analysis principles were applied during the post-implementation phase, which occurred in the subsequent three months. Data analysis was conducted using SPSS 23.0 with a significance of p-value ≤0.05.

**Figure 2:**
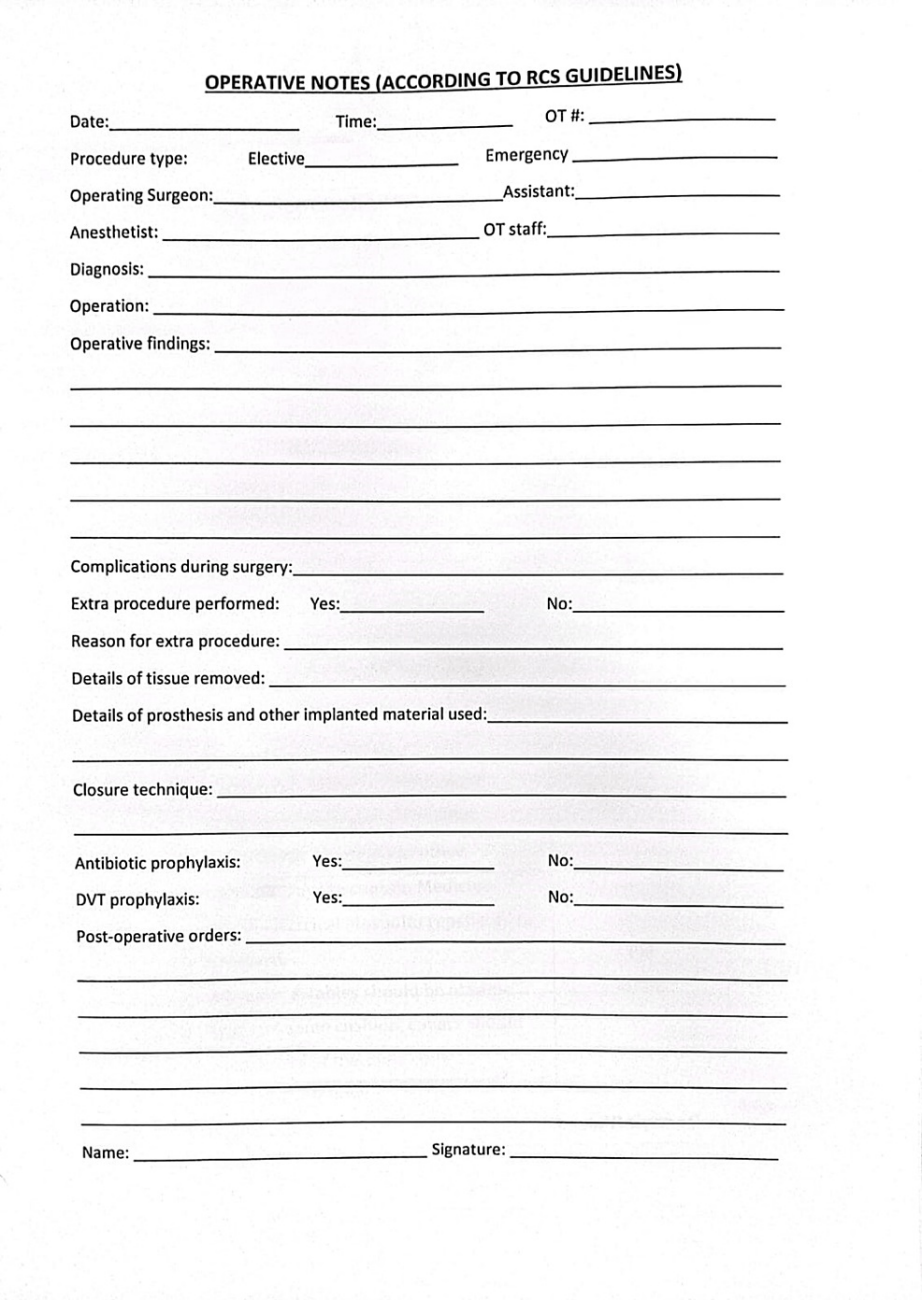
Operative notes following the implementation of RCS guidelines. RCS: Royal College of Surgeons.

During the post-implementation phase, a dedicated operative notes format aligned with the RCS guidelines was developed and put into practice. This enhancement aimed to improve the quality and adherence of operative notes to the established guidelines (Figure 2).

## Results

A thorough analysis of 150 operative notes was conducted, with 79 notes scrutinized before the implementation of RCS guidelines and 71 notes reviewed after the guidelines' introduction. The findings unveiled remarkable improvements across various critical facets of operative note documentation following the guideline implementation. Prior to RCS guideline adoption, 56 (70.8%) operative notes included peri-operative findings, either in the operative diagnosis or as a subheading in procedure notes. After the guideline implementation, this percentage soared to 68 (95.7%), signifying a significant enhancement in capturing essential peri-operative details. Pre-implementation, operative diagnosis was present in 65 (82.3%) notes. However, following RCS guideline implementation, this critical component was consistently included in 69 operative notes (97.2%), ensuring crystal-clear and comprehensive patient records. In the pre-implementation phase, the names of the anesthetist 49 (62%), operative staff, and assistant were present in 44 (56%) operative notes, while the operating surgeon's name was included in 67 (84.8%). After guideline implementation, these numbers surged to 68 (95.8%) for the anesthetist, 69 (97.1%) for the operative staff, and 69 (97.1%) for the operating surgeon, guaranteeing proper acknowledgment of the entire medical team. Before guideline implementation, 36 (45.6%) mentioned the operation type (e.g., emergency/elective), 69 (87.3%) noted the operation date and 39 (49.4%) indicated the operation time. In contrast, post-implementation, 67 (94.4%) operative notes consistently included operation type, date, and time, offering a comprehensive overview of each surgical procedure.

Pre-implementation, a mere 17 (21.5%) operative notes documented complications, while 11 (13.9%) notes mentioned any additional procedures and nine (11.4%) notes reported tissue alterations during surgery. Post-implementation, these percentages surged to 66 (92.9%) notes for complications, 64 (90.1%) notes for additional procedures, and 60 (84.5%) notes for tissue alterations, ensuring a more detailed and precise account of surgical events. Pre-implementation, 56 (70.8%) operative notes indicated prosthesis usage, with 11 (13.9%) notes detailing closure techniques. Post-implementation, these aspects were consistently recorded in 65 (91.5%) notes for prosthesis use and 66 (92.9%) notes for closure techniques, substantially enhancing the comprehensiveness of surgical records. Post-implementation, 68 (95.8%) operative notes included information on whether DVT prophylaxis was administered to patients, whereas 15 (18.9%) were documented pre-implementation. Additionally, the practice of "time out" was introduced in 68 (95.8%) operative notes post-implementation, reinforcing patient safety measures. Pre-implementation, 62 (78.5%) operative notes included detailed post-operative orders, while 65 (82.3%) notes featured signatures. Following guideline implementation, these figures improved, with 70 (98.6%) notes containing post-operative orders and 69 (97.2%) notes including signatures (Table 1).

**Table 1:** Pre- and post-implementation data. DVT: Deep vein thrombosis.

Categories	Pre-Implementation (n=79)	Post-Implementation (n=71)	P-value
Peri-operative Findings	56 (70.8%)	68 (95.7%)	0.001
Operative Diagnosis	65 (82.3%)	69 (97.2%)	0
Team Information			
Anesthetist's name	49 (62%)	68 (95.8%)	0.032
Operative staff's names	44 (56%)	69 (97.1%)	0.067
Operating surgeon's name	67 (84.8%)	69 (97.1%)	0
Operational Details			
Operation type mentioned	36 (45.6%)	67 (94.4%)	0.037
Date of operation	69 (87.3%)	67 (94.4%)	0
Time of operation	39 (49.4%)	67 (94.4%)	0.023
Complications and Procedures			
Complications mentioned	17 (21.5%)	66 (92.9%)	0.193
Extra procedures mentioned	11 (13.9%)	64 (90.1%)	0.233
Tissue alterations mentioned	9 (11.4%)	60 (84.5%)	0.169
Prosthesis and Closure			
Prosthesis use mentioned	56 (70.8%)	65 (91.5%)	0
Closure technique details mentioned	11 (13.9%)	66 (92.9%)	0.321
DVT Prophylaxis and Time Out			
DVT prophylaxis mentioned	15 (18.9%)	68 (95.8%)	0.36
"Time out" mentioned	7 (8.9%)	68 (95.8%)	0.558
Postoperative Orders and Signatures			
Postoperative orders mentioned	62 (78.5%)	70 (98.6%)	0.008
Signatures included	65 (82.3%)	69 (97.2%)	0

## Discussion

The comprehensive analysis of 150 operative notes, comprising 79 notes pre-implementation and 71 notes post-implementation of RCS guidelines, has yielded substantial insights into the enhancement of operative note documentation practices. In the pre-implementation phase, the responsibility for writing procedure notes was shared among postgraduate trainees of the department, leading to variability and potential medico-legal implications. After adopting RCS-prescribed surgical notes, a designated team member (Specialist / Trainee Registrar) ensured correctness and completeness, aligning with recognized guidelines to minimize medico-legal risks. The significant enhancement in compliance rates observed in our study reflects a multifaceted approach to improving operative note documentation practices. While introducing the RCS-aligned operative notes proforma was a pivotal component, it was complemented by an educational intervention that aimed to create awareness and understanding among surgical staff regarding the importance of meticulous record-keeping and adherence to guidelines. This intervention involved in-depth discussions of the deficiencies identified in the pre-implementation phase, emphasizing the impact on patient care. Furthermore, we conducted training sessions to ensure that surgical staff were well-versed in using the new proforma and understood its significance in capturing peri-operative findings. The iterative process of feedback and refinement was instrumental in making the proforma more user-friendly. This holistic approach, combining a standardized proforma with educational initiatives and feedback mechanisms, collectively contributed to the observed improvements in compliance rates and the inclusion of vital peri-operative details in operative notes. These findings underscore the critical role of standardized guidelines in promoting meticulous record-keeping and, consequently, patient safety and care continuity. The remarkable increase from 56 (70.8%) to 68 (95.7%) in the inclusion of peri-operative findings in operative notes post-implementation of RCS guidelines is a noteworthy achievement. This improvement ensures that essential peri-operative details are consistently captured, contributing to a more comprehensive understanding of the patient's surgical journey. These findings align with international literature emphasizing the significance of documenting peri-operative information for improved patient care [8]. It is reassuring to observe that both pre- and post-implementation phases demonstrated uniform documentation of operative interventions in all 150 operative notes. This consistency underscores the commitment to maintaining the continuity of care, ensuring that essential procedures are well-documented. This practice resonates with studies highlighting the importance of consistent documentation of operative interventions [9]. The transition from 65 (82.3%) to 69 (97.2%) inclusion of operative diagnosis in operative notes post-implementation signifies a significant stride towards maintaining crystal-clear and comprehensive patient records. This change guarantees that critical diagnostic information is consistently present, facilitating effective patient management and communication among healthcare providers. International studies have emphasized the essential role of operative diagnosis documentation in surgical records [10]. The substantial improvement in acknowledging the medical team members in operative notes post-implementation, with percentages rising to 69 (97.2%), is commendable. This change ensures proper recognition of the entire team's contributions, enhancing collaborative patient care. The adoption of standard operative procedure notes not only enhances the documentation process but also has a direct and positive impact on patient care. It improves teamwork, transparency, accountability, and the overall quality and safety of surgical procedures, all of which ultimately contribute to better patient outcomes and care. International research has highlighted the importance of team information documentation for transparency and accountability in surgical procedures [11]. The shift from a lack of consistent recording of operation type, date, and time pre-implementation to a uniform inclusion of these details post-implementation is pivotal. This standardization provides a comprehensive overview of each surgical procedure, aiding in better patient care coordination and scheduling. Similar improvements in operational details have been noted in studies from various healthcare settings [12]. The substantial increase in the documentation of complications from 17 (21.5%) to 66 (93%), additional procedures from 11 (14%) to 64 (96.1%), and tissue alterations from 9 (11.4%) to 60 (84.5%), post-implementation ensures a more detailed and precise account of surgical events. This enhancement in documentation is pivotal for monitoring patient progress and addressing any post-operative concerns. Studies have shown that comprehensive documentation of complications and procedures is vital for clinical decision-making [13]. After implementation, there was a marked improvement in the consistent documentation of prosthesis usage at 65 (91.5%) and closure techniques at 66 (93%), compared to their respective percentages before implementation. Such detailed records are crucial for managing post-operative care and any potential future procedures. The significance of noting prosthesis use and closure techniques has also been underscored in international studies [14].

The post-implementation incorporation of DVT prophylaxis documentation, evidenced by 68 (95.8%) instances, and the adherence to the "time out" procedure, also at 68 (95.8%), underscores the commitment to enhancing patient safety. This standardization contributes to reducing the risk of complications and improving patient outcomes. Literature has emphasized the importance of DVT prophylaxis and "time out" practices in surgical procedures [15]. The improvement in the documentation of postoperative orders noted in 70 (98.6%) instances and signatures, reflected in 69 (97.2%) instances post-implementation, signifies a dedication to meticulous documentation of patient care plans. Comprehensive documentation of postoperative orders and signatures is consistent with international guidelines and best practices.

The adoption of standard operative procedure notes improved patient care by ensuring that crucial details of surgical procedures were consistently documented. This enhanced documentation contributed to better clinical decision-making, patient safety, and care continuity, ultimately improving patient outcomes and overall healthcare quality.

Limitations

This study has a few limitations that should be considered when interpreting its findings. First, the reasons for non-compliance with RCS guidelines were not explored, and the rates of non-compliance were not quantified, limiting our understanding of the underlying factors contributing to non-compliance. Second, resource constraints, including limited staff availability and equipment, may have introduced selection bias, affecting data collection and analysis. These constraints may have influenced the selection of operative notes, potentially skewing the representation of diverse documentation practices. Lastly, the study was conducted in a single healthcare center, which may limit the generalizability of the findings to other settings. Additionally, the relatively small sample size of 150 operative notes may not fully represent the diversity of practices in operative note documentation. Further investigation with larger samples is warranted to assess the long-term sustainability and lasting impact of RCS guidelines on operative note quality.

## Conclusions

The implementation of RCS guidelines not only refined the content of operative note documentation but also addressed the practical aspects of their application in surgical settings. The guidelines were accompanied by the developing of a user-friendly operative note proforma and educational interventions to ensure their effective use. This comprehensive approach resulted in significant improvements in the documentation of peri-operative findings, diagnosis, team information, operational details, complications, procedures, prosthesis, closure, DVT prophylaxis, "time out," postoperative orders, and signatures. These enhancements align with international literature emphasizing the importance of standardized guidelines in elevating healthcare quality. The study underscores the significance of not only what the guidelines recommend but also how they are implemented in practice, highlighting the critical role of ongoing education and audits in sustaining these gains. Ultimately, adopting improved guidelines has promoted patient safety and care continuity in the surgical setting.
